# Tuberculosis in post-contact Native Americans of Brazil: Paleopathological and paleogenetic evidence from the Tenetehara-Guajajara

**DOI:** 10.1371/journal.pone.0202394

**Published:** 2018-09-05

**Authors:** Lucélia Guedes, Lauren Hubert Jaeger, Andersen Liryo, Claudia Rodrigues-Carvalho, Sheila Mendonça de Souza, Alena Mayo Iñiguez

**Affiliations:** 1 Laboratório de Biologia de Tripanosomatídeos, Instituto Oswaldo Cruz, Fundação Oswaldo Cruz, Rio de Janeiro, Brasil; 2 Escola Nacional de Saúde Pública Sergio Arouca, Fundação Oswaldo Cruz, Rio de Janeiro, Brasil; 3 Programa de Pós-graduação em Arqueologia, Museu Nacional/UFRJ, Rio de Janeiro, Brasil; 4 Setor de Antropologia Biológica, Departamento de Antropologia, Museu Nacional/UFRJ, Rio de Janeiro, Brasil; 5 Departamento de Endemias Samuel Pessoa, Escola Nacional de Saúde Pública Sergio Arouca, Fundação Oswaldo Cruz, Rio de Janeiro, Brasil; Ben-Gurion University of the Negev, ISRAEL

## Abstract

Tuberculosis (TB) has been described in Native American populations prior to the arrival of European explorers, and in Brazilian populations dating from the Colonial Period. There are no studies demonstrating TB infection in native Brazilians, and the history and epidemiological scenario of TB in Brazil is still unknown. The aim of this study was to verify the presence of TB infection among the native Tenetehara-Guajajara population from Maranhão State, Brazil, 210 ± 40 years ago. A Tenetehara-Guajajara skeleton collection was submitted to paleopathological analysis, and rib bone samples (n = 17) were used for paleogenetic analysis based on *Mycobacterium tuberculosis* complex (MTC) targets. Porotic hyperostosis and *cribra orbitalia* were found in 10 and 13 individuals, respectively. Maternal ancestry analysis revealed Native American mtDNA haplogroups A and C1 in three individuals. Three samples showed osteological evidence suggestive of TB. *kat*G and *mtp*40 sequences were detected in three individuals, indicating probable TB infection by two MTC lineages. Tuberculosis infection in the Tenetehara-Guajajara population since the 18th century points to a panorama of the disease resulting, most probably, from European contact. However, the important contribution of African slaves in the population of Maranhão State, could be also considered as a source of the disease. This study provides new data on TB during the Brazilian Colonial Period. This is the first report integrating paleopathological and paleogenetic data for the study of TB in Brazil.

## Introduction

Tuberculosis is an infectious disease caused by bacteria of the *Mycobacterium tuberculosis* complex (MTC), which comprises *Mycobacterium tuberculosis sensu stricto*, *M*. *africanum*, *M*. *canettii*, *M*. *bovis*, *M*. *caprae*, *M*. *microti*, *M*. *pinnipedi*, and *M*. *mungi* [1,2]. *Mycobacterium tuberculosis* complex emerged about 70,000 years ago and accompanied migrations of anatomically modern humans out of Africa [3]. Previously, it was believed that human TB originated in other animals, adapting to humans during the Neolithic, but current phylogenetic analyses suggest that strains adapted to other animals diverged from human strains before that period [4,5], and there is an evolutionary distance of at least 20,000 years between *M*. *tuberculosis sensu stricto* and other animal strains [6].

Paleopathological evidence described in pre-Columbian populations of Peru, Chile [7,8], and Venezuela [9] suggests that tuberculosis existed in America before the arrival of Columbus [10,11]. Genetic study has revealed MTC in human remains from Peru [12,13], Chile [14], and Colombia [15]. Evidence of *Mycobacterium tuberculosis* complex infection caused by ancient strains most closely related to those from sea lions was recently found in Peruvian mummies [16]. In Brazil, ancient MTC DNA (aDNA) has been detected from the Colonial Period of Rio de Janeiro, showing TB infection in individuals of predominantly European ancestry [17], as well as in African slaves [18]. There are no reports of TB infection in pre-Columbian native communities from Brazil.

We used paleogenetic analysis to investigate presence of TB infection in Native American Tenetehara-Guajajara skeletal remains dating from 210 ± 40 years ago, post-contact with Europeans and Africans. Study of a post-contact native group with bone lesions suggestive of TB allowed investigation of biological and cultural influences on disease processes in those Brazilian communities.

## Material and methods

### Ethics statements

The Tenetehara-Guajajara skeletal remains are held in the collection of the Biological Anthropology Section (*Setor de Antropologia Biológica*-SABMN) of the National Museum of the Federal University of Rio de Janeiro (*Museu Nacional/Universidade Federal do Rio de Janeiro* -MN/UFRJ). Rib samples (n = 17), SABMN00699-SABMN00707, SABMN00709-SABMN00711, SABMN00713-SABMN00715, and SABMN00717- SABMN00718, were supplied for the SABMN/MN/UFRJ for analysis at the paleogenetic laboratory of LABTRIP/IOC/Fiocruz. All necessary permits were obtained for the study, which complied with all relevant regulations.

### The Tenetehara-Guajajara

The Tenetehara-Guajajara is an extant Tupi speaking native Brazilian community, one of the first to be contacted during the Colonial Period. Tenetehara means "be intact" [19]. Currently, the population of 24,428 individuals [20] is distributed in villages in Pindaré-Gurupi River basin, Maranhão State, Brazilian Amazonia. Their economy was traditionally based on growing corn and cassava, supplemented with hunting and fishing. Its main cultural ceremonies were honey and corn festivals, which accompanied the harvest season [21]. They were first encountered by French explorers in 1612, and, in 1616, Portuguese expeditions for gold began a period of war and slavery [22]. In 1653, catechizing of the Tenetehara by the Jesuits initiated the period of coexistence with Europeans [23]. Their history of conflict, slavery, and domination lasted for more than four centuries, but some small groups succeeded in escaping to distant settlements in the Pindaré-Gurupi River basin, where they lived for decades [24,25].

In 1945, when the isolated Tenetehara no longer existed, Dr. Pedro Lima travelled to the Pindaré-Gurupi River villages to study the bio-anthropology and health of the community [26]. During his ethnographic fieldwork, he exhumed skeletons from cemeteries of Kamirang and Januária villages, with the full consent of the Tenetehara leaders. Twenty-one complete skeletons of adults and children were recovered and studied at the MN/UFRJ. Recent analysis revealed that the individuals lived during the end of Colonial Period and the beginning of Brazilian Empire. The sample designated SABMN00718 is from 210 ± 40 years BP (GEOCHRON MA GX31824-AMS C^13^ corrected), and SABMN00717 is from 140 ± 30 BP (BETA 291714-AMS, C^13^ corrected) [27].

### Review of skeletal and dental characteristics

The original series of remains was numbered from SABMN00699 to SABMN00719 in the book of the Biological Anthropology Section (MN/UFRJ). Skeletons were examined to i) provide general descriptions [28]; ii) identify/confirm signs of possible infectious disease [29,30]; iii) provide contextual data for paleo-epidemiological interpretation; and iv) select bones for the paleogenetic analysis.

Most of the major bones were available for examination, although the small and spongy bones were lost, because of taphonomic processes in the humid tropical location of the cemetery. Teeth were still articulated and were examined *in situ*. Ribs were chosen for analysis because of their direct association with lung disease and acceptable state of preservation.

Criteria for bone samples to be submitted to aDNA analysis were i) rib samples only from adult/subadult skeletons of both sexes; ii) only one rib from each individual, either left or right; and iii) specimens with pathological signs were not used for the paleogenetic analysis.

### Paleogenetic analysis

#### Precautions to avoid contamination

Precautions were taken to avoid contamination by modern DNA and cross-contamination, including use of protective clothing, gloves, head covering, masks, and sterile instruments and equipment. We implemented standard aDNA procedures to avoid aDNA degradation, contamination from modern DNA, and cross-contamination during the paleogenetic analysis as described [31–33]. The preparation of samples, aDNA extraction, and PCR were performed in the Paleogenetic Laboratory (LABTRIP/IOC/FIOCRUZ) in an isolated environment, in facilities exclusively dedicated to aDNA research. Hybridization assay, positive control PCRs for constructing DNA probes, electrophoresis, DNA sequencing, and sequence analysis were conducted at main laboratory (LABTRIP, IOC/FIOCRUZ). These laboratories are separated by 500 m. All work surfaces and equipment were treated with 1% sodium hypochlorite and UV irradiated. All reagents were separated into single-use aliquots. Extraction blank controls were processed in parallel with samples (1 blank for each 6 samples), and PCR negative controls were always included. The authenticity criteria included the absence of detectable PCR products (pPCR) in sediment removed from the surface of bones (archaeological site controls), extraction blank and PCR negative controls, and pretreatment by reconstructive polymerization (PR) and Whole Genome Amplification (WGA- REPLI-g, Qiagen). PCR positive controls were not included in the Paleogenetic Laboratory and were only applied in the main laboratory (LABTRIP, IOC/FIOCRUZ) as a technical requirement for the construction of MTC probes. Two and six genetic targets were used for MTC aDNA hybridization and detection/genotyping, respectively. Human DNA was analyzed in parallel with the MTC aDNA detection.

#### aDNA extraction

Seventeen ribs were provided by the biological anthropology section (MN/UFRJ) for paleogenetic analysis (Table 1). Exogenous DNA was removed from samples by exposing the surface to UV light for 15 min on all sides and subsequently removing the surfaces [34]. Bones were submitted to mill trituration with liquid nitrogen. About 200 mg of bone powder was used for aDNA extraction with the QIAmp DNA Investigator kit (Qiagen) according to the manufacturer’s instructions with the following modifications: Protein digestion was performed by adding 30 μL of proteinase K (Invitrogen) at 20 mg/μl, and the incubation period of elution buffer was increased to 10 minutes at room temperature and final centrifugation at 17,000 xg for 2 minutes. The concentrations of aDNA were estimated at 260 nm absorbance on a spectrophotometer NanoDrop^™^ 1000. Sediment samples removed from the first cleaning of bones were used as controls for the archaeological site and submitted to MTC paleogenetic procedures.

**Table 1:** Bio-anthropological analysis of Tenetehara-Guajajara individuals from the Biological Anthropology collection (MN/UFRJ).

Village	Individual	Sex ^a^	Age	Sample
**Kamirang**	SABMN00699	M	Adult	Right rib
	SABMN00700	F	Young	Right rib
	SABMN00701	F	Adult	Right rib
	SABMN00702	M	Adult	Right rib
	SABMN00703	M	Adult	Left rib
	SABMN00704	M	Adult	Left rib
	SABMN00705	U	Child	Left rib
**Januária**	SABMN00706	F	Adult	Left rib
	SABMN00707	M	Young	Left rib
	SABMN00709	F	Adult	Right rib
	SABMN00710	M	Adult	Right rib
	SABMN00711	M	Adult	Right rib
	SABMN00713	U	Child	Right rib
	SABMN00714	U	Child	Right rib
	SABMN00715	F	Adult	Right rib
	SABMN00717	F	Adult	Left rib
	SABMN00718	M	Adult	Left rib

^a^ According to [28]. Adults >20 years; Young 14–20 years; Child 6–13 years. Abbreviations: M, male; F, female; U, undetermined.

#### MTC aDNA hybridization

Dotblot and aDNA hybridization procedures were conducted as described elsewhere [17]. Human DNA was used as negative control. DNA probes of 93 and 113 bp, corresponding to MTC molecular targets IS6110 and IS1081, respectively [35,36], were prepared by PCR. DNA from four MTC type strains were used as hybridization positive controls for IS6110 and IS1081 probes: *M*. *tuberculosis* T92 (ATCC27294^T^), *M*. *tuberculosis* H37Rv (ATCC27294^T^), *M*. *bovis* BCG (ATCC19210^T^) and *M*. *africanum* T85 (ATCC25420^T^). The pPCRs were purified by a GFX PCR DNA and Gel Band Purification kit (GE HealthCare) and directly sequenced using an ABI BigDye Terminator kit (Applied Biosystems) according to the manufacturer’s protocol, with analysis in both directions, on an ABI 3730 (Applied Biosystems) automated sequencer. BioEdit v. 7.0.4 (Department of Microbiology, North Carolina State University, USA), and Lasergene Seqman v. 7.0.0 (DNASTAR, Madison, WI, USA) were used for editing and sequence analysis. Sequencing and sequence analysis were performed to confirm MTC molecular targets. Probes were labeled by chemiluminescence using Gene Images Alkphos Direct Labeling and Detection Systems (Amersham) as described elsewhere [17].

#### MTC aDNA amplification

To confirm MTC infection and exclude false positive or environmental bacteria contamination, PCRs using MTC targets IS6110 and IS1081 were conducted on archaeological samples, following conditions and primers described [35, 36], using Platinum *Taq* DNA Polymerase High Fidelity (Invitrogen). Additional MTC genotyping markers were applied, as described (Table 2). These molecular targets are widely used in aDNA studies to determine MTC species or lineages implicated in TB infection. In cases of negative PCR results, aDNA was submitted to reconstructive polymerization (RP) [37], using Platinum *Taq* DNA Polymerase High Fidelity (Invitrogen), and whole genomic amplification (WGA) (S1 Table). Reconstructive polymerization pretreatment has been used for reconstructing and amplifying total aDNA [17]. WGA was also conducted, since, in contrast to RP, the DNA is amplified based on short and random sequence primers, avoiding bias [38]. PCR products were analyzed by electrophoresis in agarose gels and visualized under UV light. Due to the weak bands obtained, pPCRs were purified with MiniElute Gel Extraction Kit (Qiagen). PCR results were replicated at least twice. pPCRs were submitted to cloning using pGEM^®^-T and pGEM^®^-T Easy Vector Systems kit (Promega) following manufacturer’s instructions, with least three clones produced. Sequencing and sequence analysis were performed as described above. MTC sequences were submitted to GenBank.

**Table 2:** Targets used to amplify MTC aDNA from Tenetehara-Guajajara skeletal remains from Maranhão State, Brazil.

Target	PCR product (bp)	TB polymorphism	References
*oxy*R	110/94	*oxy*R285	[36]
*kat*G	142	*kat*G203	[39]
mtp40	152	mtp40	[39]
*pnc*A	117/96	*pnc*A57	[36]

#### Human mtDNA amplification and sequencing

The hypervariable segment I (HVS-I) of the mtDNA was used as target to determine human ancestry. Four primer pairs were used: L16070/H16259 [17], L16209/H16410 [40], L16268/H16498 [41] and L16234: 5’-CACATCAACTGCAACTCCAAA-3’ and H16422: 5’-ATTGATTTCACGGAGGATGG-3’, designed in this study using PRIMER3 (http://www.bioinformatics.nl/cgi-bin/primer3plus/primer3plus.cgi/). PCR procedures, using Platinum *Taq* DNA Polymerase High Fidelity (Invitrogen), were replicated at least twice and pPCRs directly sequenced or/and cloned. Cloning, sequencing, and sequence analysis were performed as described above. Cambridge Reference Sequence (CRS, GenBank: NC12920, [42]) was used to identify mtDNA haplotypes, and sequences obtained were compared with mtDNA database of the paleogenetic lab staff to identify and discard contaminated sequences. The Tenetehara-Guajajara mtDNA sequences were submitted to GenBank.

## Results and discussion

### Relevant skeletal and dental data

Skeletal remains of 17 adults/subadults of the Tenetehara collection were analyzed, comprising eight males, six females, and three undetermined (Table 1). The long bones were gracile and the stature was short. The results were consistent with the description in ethnographic literature and the anthropometric descriptions for contemporaneous Tupi groups in Brazilian Amazonia. According to Lima [43], anthropometric studies of living Tenetehara showed the smallest stature among the Tupi groups (1.4–1.5 m). Our findings of small stature and the extremely frail and delicate long bones, in both males and females, pointed to severe malnutrition and underdevelopment, consistent with descriptions of poor health suffered by the Tenetehara over the centuries [25]. Individuals living in the mid-20th century experienced severe infections such as malaria and pneumopathies, as well as other conditions including stunted growth [43].

**Fig 1:**
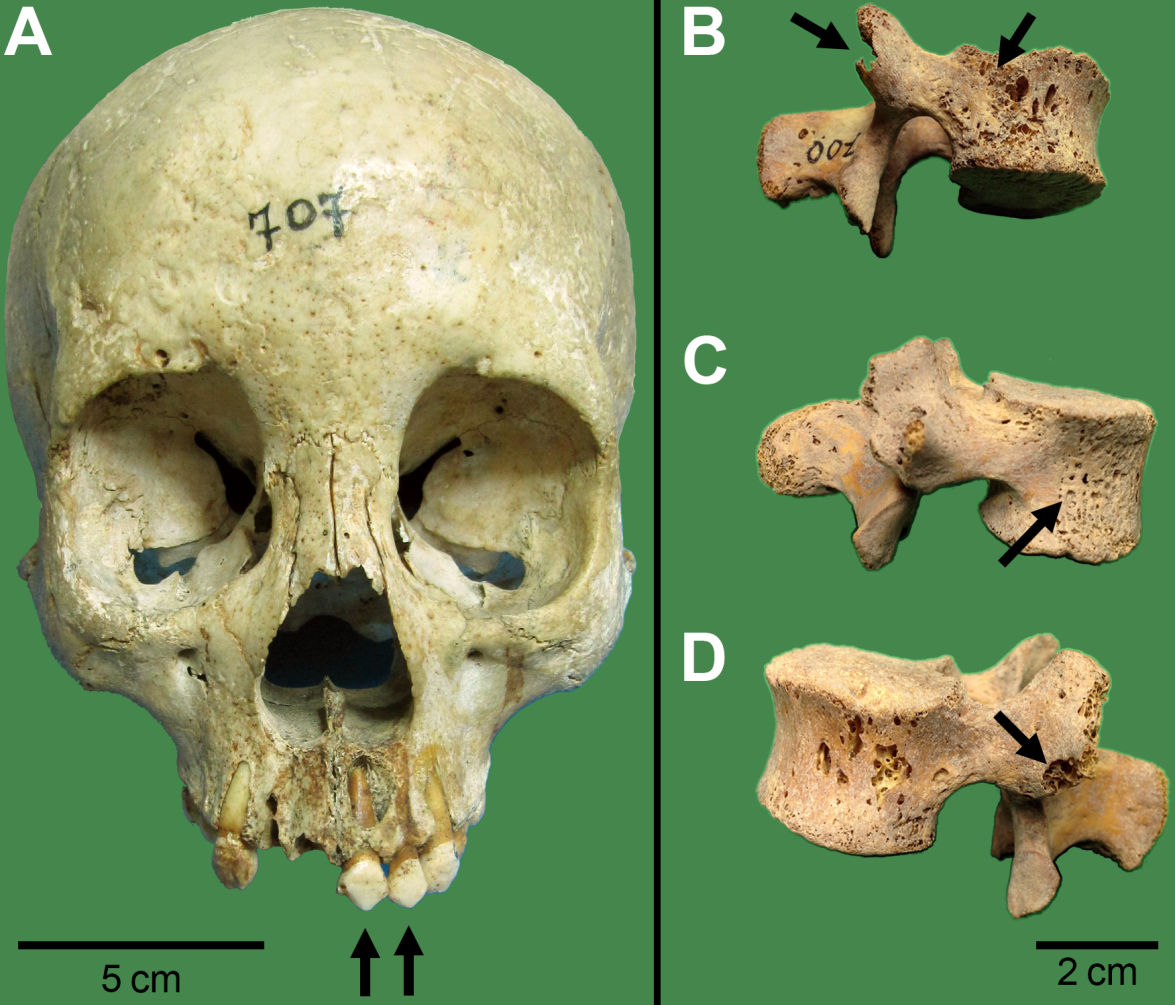
Intentional dental modification and bone anomalies suggestive of TB in skeletal remains of Tenetehara-Guajajara. (A) Frontal view of the skull of individual SABMN00707. The arrows indicate intentional dental mutilation of the upper incisors, the so-called *piranha* teeth. (B-D) Lumbar vertebrae of individual SABMN00700. (B) Left arrow indicates bone loss at the upper right zygapophysis of vertebrae. Right arrow indicates porotic changes of the vertebral body suggestive of TB. (C) Porotic changes suggestive of TB at the vertebral body (arrow). (D) Bone loss at the upper left zygapophysis of vertebrae (arrow).

The rounded mongoloid skull was consistent with the description of most Native Amazonian communities, and dental loss and caries were observed in most individuals. Intentional dental modification was described in contemporary Tenetehara-Guajajara by Lima [43] and confirmed in the present study. So-called *piranha* teeth, with both angles of the four upper incisors cut off with a blade [43], were present in individuals SABMN00699, SABMN00701, SABMN00704, SABMN00707, and SABMN00715 (Fig 1A). As previously suggested and discussed [43,44], this dental modification was not a cultural tradition of the Tenetehara-Guajajara, and was possibly adopted after African slave and Afro-American influence during the Colonial and Imperial Periods. African influence is especially important, considering the substantial proportion of Africans in the population of Maranhão State [21].

**Fig 2:**
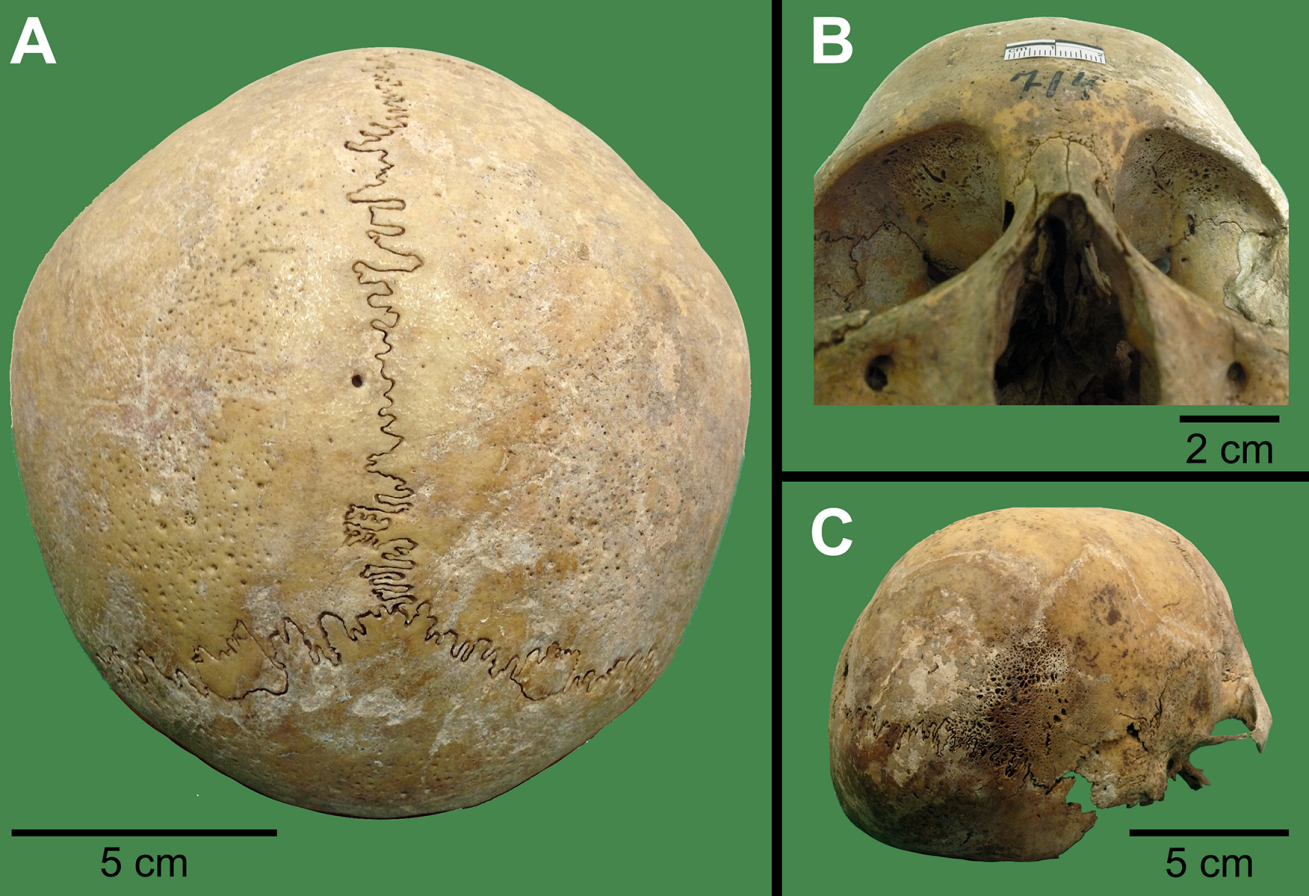
Paleopathological evidence in skeletal remains of Tenetehara-Guajajara. (A) Porotic hyperostosis in skull from individual SABMN00710. (B) Frontal view of skull from individual SABMN00714 showing *cribra orbitalia* (C). Lateral view of the skull from individual SABMN00714 with bone proliferation at the outer table.

Analysis of the joint surfaces and stress markers such as porotic hyperostosis and *cribra orbitalia* indicated trauma and underweight affecting even the young individuals, consistent with people facing extreme challenges to survival. Some developmental anomalies, such as butterfly vertebrae, and rib anomalies were observed (Fig 1B and 1C). Some bone indicators (Table 3; Fig 2) supported the hypothesis of nutritional and infectious stress [45]. Porotic hyperostosis (Fig 2A) and *cribra orbitalia* (Fig 2B) were found in 10 and 13 of the 17 individuals, respectively. Porotic hyperostosis was described by Mello et al. [46] who studied 20 Tenetehara-Guajajara skulls of adults and immature individuals, finding 16 positive for *cribra orbitalia* (Fig 2B) and porotic hyperostosis (Fig 2C). The authors, as supposed by Lima [43], suggested endemic malaria as a possible cause of anemia.

**Table 3:** Paleogenetic and paleopathological data from Tenetehara-Guajajara skeletal remains from Maranhão State, Brazil.

Village	Sample	MTC Hybridization		mtDNA Human ancestry	Bone lesions suggestive of TB and stress markers
		IS6110	IS1081		
**Kamirang**	SABMN00699	-	-		porotic hyperostosis
	SABMN00700	+	+		lithic areas in cervical and lumbar vertebrae; rib periostitis; porotic hyperostosis; *cribra orbitalia*
	SABMN00701	+	+	C1	rib periostitis; *cribra orbitalia*
	SABMN00702	+	+		porotic hyperostosis
	SABMN00703	+	+	A	*cribra orbitalia*
	SABMN00704	-	-	C1	periosteal reaction at lumbar vertebra; porotic hyperostosis; *cribra orbitalia*
	SABMN00705	-	-		porotic hyperostosis; *cribra orbitalia*
**Januária**	SABMN00706	-	-		*cribra orbitalia*
	SABMN00707	-	-		porotic hyperostosis; *cribra orbitalia*
	SABMN00709	-	-		-
	SABMN00710	-	-		porotic hyperostosis; *cribra orbitalia*
	SABMN00711	+	+		-
	SABMN00713	-	-		porotic hyperostosis; *cribra orbitalia*
	SABMN00714	-	-		*cribra orbitalia*
	SABMN00715	-	+		*cribra orbitalia*
	SABMN00717	-	+		porotic hyperostosis; *cribra orbitalia*
	SABMN00718	+	-		porotic hyperostosis; *cribra orbitalia*

Abbreviations: MTC, *Mycobacterium tuberculosis* complex; (+) positive; (-) negative.

Tuberculosis in the Tenetehara-Guajajara individuals was first suggested by Altamirano [47], who observed destructive changes in some vertebral bodies and periosteal reactions in the skeletons of adult SABMN00700, SABMN00701, and SABMN00704 (Fig 1B–1D), Although bone anomalies could be suggestive of TB, mycotic lesions must be considered.

Findings of Altamirano [47] confirmed by the present study were as follows:

1. SABMN00700: 13–14 year old female–discoloring at the internal surface of the 7th, 8th, and 9th left ribs suggestive of periostitis; small lithic lesions at the pedicles and other areas of the vertebral arches and bodies.
2. SABMN00701: 20–25 year old female–periosteal reactions in ribs and other bones plus trauma suggestive of possible infection.
3. SABMN00704: 30–35 year old male—periosteal reaction in the ventral part of the body of a lumbar vertebra.

The characteristics of the Tenetehara-Guajajara skeletons are consistent with the poor health associated with economic constraints, poverty, and social disruption described in the ethnohistorical and bio-anthropological reports, contributing to recurrent epidemic or endemic diseases such as malaria, pneumopathies, smallpox, and others [43].

### mtDNA analysis

Some fragments of HVS-I target were successfully amplified by PCR, cloned and sequenced (S1 Fig). Human mtDNA analysis identified the mtDNA haplogroup in three Tenetehara individuals (17.6%) (Table 3). The haplogroups classified were the Amerindian mtDNA macrohaplogroups A and C1 (GenBank ID KM066101-KM066103). We recovered and analyzed 183 bp of the mtDNA sequence of individual SABMN00703, which showed two of three HVS-I motifs for haplogroup A, 16223 and 16290 [48]. Cloning of pPCRs using L16209/H16410 primers confirmed 99–100% Amerindian haplotype (S1 Fig).

Sequences 353 and 239 bp in length were recovered from SABMN00701 and SABMN704, respectively (Table 3). The sequences showed the HVS-I motifs 16223, 16298, 16325, and 16327 (Table 4), which characterize haplogroup C1 [48]. Cloning of pPCRs using L16070/H16259-L16234/H16422 and L16268/H16498 primers confirmed 100% and 99–100% of haplotype identification in SABMN00701 and SABMN704, respectively (S1 Fig). Haplogroup sequences for SABMN00701 and SABMN00704 revealed 100% and 99% identity, respectively, with haplogroup C1 from Native South Americans (KC676569 [49]; JQ996071 [50]; EU095227 [51]).

**Table 4:** Human mtDNA haplogroups and HVS-I polymorphism in Tenetehara-Guajajara remains, Maranhão State, Brazil.

Samples	Nucleotide position ^a^										mtDNA Haplogroup
	1 2 9	2 0 4	2 2 3	2 9 0	2 9 2	2 9 8	3 2 5	3 2 7	3 6 2	4 9 6	
**CRS**	G	G	C	C	C	T	T	C	T	G	
**SABMN00701**	.	.	T	.	T	C	C	T	C	-	C1
**SABMN00703**	-	A	T	T	.	.	.	.	.	-	A
**SABMN00704**	-	-	-	.	T	C	C	T	C	A	C1

^a^ Prefix 16 according to Cambridge Reference Sequence (CRS)—GenBank: NC012920 [42]. Abbreviations: bp, base pair; (.), nucleotide equal to CRS; (-), not sequenced.

Our results agree with Leite et al. [27] who identified haplogroups A and C in ancient Tenetehara-Guajajara remains. Haplogroup A is the most frequently found in contemporary Brazilian Natives. The autochthonous haplogroup C1 is widely distributed among North, Central, and South Americans [52,53]. In Brazil, it was found in a native Je speaking group from the 19th century called the Botocudo [54]. In contemporary populations, the C1 haplogroup has been described in a native Karib speaking group called Arara, from Pará State [51], a neighbor to Maranhão State. It was also observed in about 70% of Amerindian descendants of a rural community of Minas Gerais State, Southern Brazil, and described in 16.7% of haplogroups from modern unrelated samples from the same region [54]. Moreover, HVS-I motifs from C1 haplogroup have been observed in other regions of the country [55].

### Tuberculosis

Positive MTC hybridization was observed in eight of 17 Tenetehara individuals, including six and seven with the IS6110 and IS1081 targets, respectively (Table 3). Five samples showed positive hybridization results with both targets.

SABMN00700 and SBMN00701, which showed periosteal reactions and indications of infective lesions were positive for MTC hybridization by both IS6110 and IS1081. A third individual (SABMN00704) with rib periosteal reactions did not show positive results for MTC aDNA hybridization. Seven specimens positive for MTC hybridization demonstrated porotic hyperostosis and *cribra orbitalia* (Table 3).

All positive pPCR including those of unexpected length were submitted to nucleotide sequencing. The MTC sequencing that provided low quality or no sequences were excluded. Nucleotide sequencing confirmed PCR results for *mpt*40 and *kat*G targets in three individuals. PCR exhibited absence of amplification for all molecular targets in negative controls, even after application of RP and WGA.

RP and WGA increased the aDNA concentration (S1 Table) and probably aDNA quality, make PCR amplification more efficient. These approaches have been applied by us, and others, in paleogenetic studies [17,31,32, 56–58]. We chose to use the WGA based on MDA technology which uses a Phi29 polymerase which has 3'→5' exonuclease activity and a higher fidelity during replication compared to *Taq* DNA polymerase (qiagen.com). However, RP and WGA could produce chimeras in non-homogenous aDNA extracts. Forst and Brown [59] attested that WGA does not provide advantage in studies of MTC aDNA in human skeletons. However, previously, Forst [60] verified the success WGA application in archaeological samples to detect the MTC complex [59,60]. The author stated that probably the efficiency of technique depends on the age and preservation of sample. Accordingly, the experience with WGA application seems to be divergent. We obtained good increases on aDNA concentration, but indistinct tendency in PCR target detection (S1 Table). Since all positive pPCR were cloned and sequenced, if chimeras resulting of RP or WGA treatment were produced, they would be easily identified during the sequence analysis. The aDNA sequences from this study showed 100–98% of identity with the molecular targets applied, so, there is no doubts on the positive results achieved.

**Fig 3:**
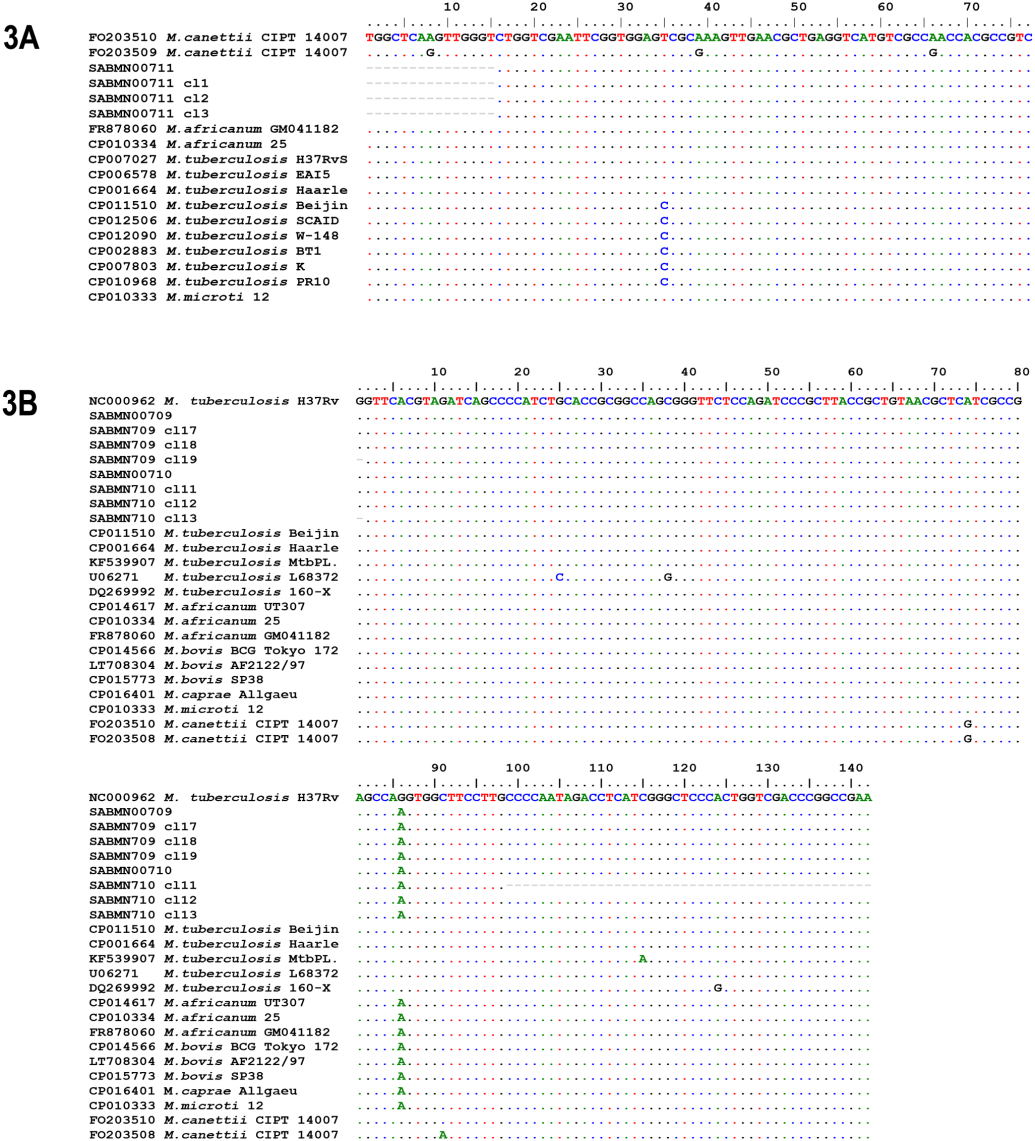
*Mycobacterium tuberculosis* complex alignments of *mtp*40 and *kat*G sequences of Tenetehara-Guajajara individuals. 3A: *mtp*40 sequence and clones from sample SABMN00711 with *M*. *canettii* (GenBank FO203510) as reference sequence. All *M*. *canettii*, *M*. *africanum*, and *M*. *microti* sequences available in GenBank were included. The two type sequences from *M*. *tuberculosis* are shown with the T35C polymorphism. 3B: Alignment of *kat*G sequences and clones of Tenetehara-Guajajara individuals SABMN00709 and SABMN00710 using *M*. *tuberculosis* (GenBank NC000962) as reference sequence. All *M*. *caprae*, *M*. *microti*, and *M*. *canettii* sequences available in GenBank were included.

We obtained *mtp*40 sequence fragment (Fig 3A) from individual SABMN00711 (GenBank: KY039569) and the complete sequence of the *kat*G target (GenBank: MF773496-97) from SABMN00709 and SABMN00710 (Fig 3B).

The *mtp*40 sequence demonstrated 100% or 98% identity with all *M*. *tuberculosis* sequences available. The SABMN00711 sequence showed T at position 35 (Fig 3A) identical to H37R (GenBank: CP007027), EAI (GenBank: CP006578), and Haarlem (GenBank: CP001664) strains. In contrast to the Beijing (GenBank: CP011510) strain, which exhibits the T35C SNP (Fig 3A). Maximum identity was observed with other MTC strains, including *M*. *africanum* 25 (GenBank: CP010334), *M*. *canettii* CIPT140070017 (GenBank: FO203510), and *M*. *microti* 12 (GenBank: CP010333). *mtp*40 is absent in some MTC strains, including *M*. *bovis*, *M*. *caprae*, and some *M*. *tuberculosis* [16]. Nevertheless, it has been shown to be specific for *M*. *tuberculosis* and *M*. *africanum* [61,62]. The short sequence amplified did not allow confirmation of the MTC species/lineage involved in SABMN00711 infection, but strongly indicated infection by these species rather than by *M*. *bovis*. Fletcher et al. [39] detected the *mtp*40 gene in three 18th century Hungarian mummies, excluding *M*. *bovis* infection. The SABMN00711 individual was positive for IS aDNA hybridization, but no TB or nonspecific bone lesions were observed. The present study confirmed infection by pathogenic MTC *Mycobacteria*.

The *kat*G sequences from samples SABMN00709 and SAMMN00710 showed the SNP A74G that discriminates *M*. *canetti* and G86A strains from all *M*. *tuberculosis* and *M*. *canetti* sequences. G86A SNP is present in *M*. *africanum*, *M*. *bovis*, *M*. *caprae*, and *M*. *microti* strains and corresponds to the ACT (Thr) 203 codon described (in reverse position) by Fletcher et al. [39]. The *kat*G 203 target has been used to identify MTC subspecies. Huard et al [63] classified MTC species in four principal genetic groups (PGG), with ACT *kat*G 203 present in PGG1a, including *M*. *africanum* subtype Ia, *M*. *microti*, *M*. *caprae*, and *M*. *bovis*. Other PPGs with ACC *kat*G 203 include 1b: *M*. *canettii*, *M*. *tuberculosis*, *M*. *africanum* subtype Ib; 2: *M*. *tuberculosis*; 3: *M*. *tuberculosis*. Fletcher et al [39] genotyped PGG 2 and 3 in 18th century human remains found in a Hungarian crypt by *kat*G analysis. In the present study, MTC strain PPG 1a was identified, suggesting *M*. *africanum* subtype Ia or *M*. *bovis* infection in samples SABMN00709 and SABMN00710, rather than *M*. *tuberculosis* strains. Both these individuals were negative for IS aDNA hybridization, and only SABMN00710 had both porotic hyperostosis and *cribra orbitalia*, bone evidence of poor health.

Tuberculosis genotyping revealed two MTC species/lineages affecting the Tenetehara-Guajajara population. The *mtp*40 results pointed to *M*. *tuberculosis* or *M*. *africanum* strains, while *kat*G ruled out *M*. *tuberculosis* strains, suggesting *M*. *africanum* and *M*. *bovis* members. This multiple infection possibility reflects the post-contact scenario blending cultures and epidemiological backgrounds of Europeans and Africans.

Jong et al. [64] discussed possible reasons that *M*. *africanum* has not become established outside of West Africa. They point out that, even with the massive migration to the Americas during slave trade, the diseased either did not survive the journey, or, if it did, was outcompeted in the New World by *M*. *tuberculosis* [64]. Host preference of *M*. *africanum* for ethnic West Africans was also suggested by the authors as a reason it did not become established in Native Americans or European explorers. The results presented here may show some level of *M*. *africanum* strain infection.

A previous study discussed the lack of specificity of IS6110 and IS1081 targets [65]. Our attempts to sequence other specific targets were not satisfactory, except for the *kat*G and *mtp*40 segments. This was not unexpected, due to the highly degraded aDNA. Hybridization is reported to be more sensitive than PCR in aDNA detection [17], especially in highly degraded samples [66] that are better analyzed by probes that can bind to the fragmented aDNA, unlike PCR, which requires the presence of intact DNA fragments for amplification. We applied IS aDNA hybridization as a screening tool for MTC diagnosis, due to its sensitivity, with the subsequent application of a more specific approach based on PCR, cloning, and sequencing of specific MTC targets. The results confirmed MTC infection in three individuals. However, it is important to note that IS aDNA hybridization failed in screening for MTC infection, since two of three TB-positive individuals were negative by this technique. In addition, one of the three showed bone evidences of poor health with porotic hyperostosis and *cribra orbitalia* manifestations.

Finding anatomically normal bones positive for MTC aDNA has been previously described, including in skull, femur, and ribs [17,18]. Rollo et al. [67] argue that the manifestations of disease in bones are generally expressions of chronic conditions. In addition, MTC strain, as well as organs of primary focus may affect the presence and distribution of bone lesions. Either contiguous spread or bloodstream dissemination can explain MTC presence in bones, as well as pathological lesions. Periosteal reactions may be explained by blood dissemination from distant foci [68], or contiguous inflammatory responses. On the other hand, during TB infection, systemic blood dissemination may occur without visible macroscopic lesions in bones [69,70].

No detailed medical documents can be found for the Colonial Period or for Jesuits and travelers during the Empire Period in Maranhão State. Data from the former Native Protection Service (*Serviço de Proteção ao Índio—*SPI) and other Brazilian government agencies concerned with indigenous health, confirm the endemic conditions of the Tenetehara-Guajajara in the 20th century. Records describe malaria, smallpox, and syphilis, as well as TB, among other endemic and epidemic diseases. The impoverished living standards of the Tenetehara-Guajajara population following the Colonial Period contributed to health issues faced in the past century. Some of their groups succeeded in escaping slavery and extermination, migrating upstream in the Pindaré-Gurupi basin, and remaining isolated in poor conditions for almost a century.

As described by Wilbur and Buikstra [71], social disruption, forced mobility, crowding onto reservations, poor sanitation, extreme poverty, and malnutrition, with frequent exposure to pathogens, contributed to TB as a population-wide health problem. This was the case with the Tenetehara-Guajajara people since the 18th century. Tenetehara-Guajajara individuals studied here lived from the mid-18th to the mid-19th centuries in contact with Brazilian society after traditional isolation. They probably had contact with European explorers, as well as with African and/or Brazilian mestizos. In the mid-19th century, the village of Januária was colonized while Afro-Brazilian people were escaping from the Cabanagem wars in the region [24]. Cultural practices described by [43] and others confirm the contact with, and the assimilation of, African culture.

Paleogenetic studies showed TB infection in Rio de Janeiro during the 17th to 19th centuries [17,18], when poor sanitation and parasitic infections were widespread in urban areas regardless of social status [72,73]. The prevalence of TB of 53.1% in people of European ancestry demonstrated the prominent contribution of Europeans to the introduction or spread of disease in the city [17]. Additionally, 25% MTC infection among just-arrived African slaves revealed TB resulting from European contact in Africa and/or caused by native African strains [17]. The Tenetehara-Guajajara had contact with French, Portuguese, and other ethnic groups during the Brazilian Colonial Period [22,23] as well as with African-born individuals in Maranhão State, the capital of which, São Luís, was an important slave port [24,43]. Historical descriptions reported TB epidemics in Europe during the 16th and 18th centuries [74], coincident with the period of first contact and strong penetration of explorers into Tenetehara-Guajajara territory in the Pindaré-Gurupi River basin region.

Although it is well-known that TB existed in America before Columbus, possibly at a low level of endemicity [74,75], there is no paleopathological evidence of TB in pre-contact Brazilian populations. Some Native American communities may have never been exposed to infection, while others had certainly been in contact with some mycobacteria strains [11]. This study did not determine the source of Tenetehara-Guajajara infection, but we cannot rule out the possibility of African, or worldwide, strains.

Our findings suggest that the association of positive TB aDNA and bone lesions in the Tenetehara-Guajajara skeletal series is epidemiologically supported, not only by the living conditions, but by indicators of poor health condition in the remains. Cranial porotic lesions are cited among the most frequent pathological signs in ancient human skeletal collections, and have been widely accepted as probably caused by anemia, a high pathogen load, or to scurvy [76–78].

## Conclusions

In this study, we described for the first time MTC infection in a post-contact Native Brazilian population with paleopathological evidence suggestive of TB. This study provides new data supporting the occurrence of TB in the Brazilian Colonial Period. Paleopathological evidence suggested TB infection among native groups in the Pindaré-Gurupi River basin, far from the urbanized areas and farmlands created during the Colonial Period.

It is not clear whether TB infection in Tenetehara-Guajajara was caused by contact with European settlers or African slaves. They were infected by at least two MTC strains. Despite the limited number of individual remains examined, these results can have valuable impact on filling gaps in the history of TB in the Americas.

## Supporting information

S1 Table Data of aDNA concentrations of extract type, including pre-treated aDNA with RP and WGA for each sample and obtained pPCR confirmed by sequencing. (XLSX)

S1 Fig mtDNA alignment with clones generated in this study. Nomenclature of clones: The first number correspond to mtDNA PCR target (cl1-cl4) and the second to the number of clones generated. Target 1: primers L16070/H16259; 2: L16209/H16410; 3: L16234/H16422; 4: L16268/H16498.
(TIF)
