# Supplementary figures and images for: Tuberculosis in post-contact Native Americans of Brazil: Paleopathological and paleogenetic evidence from the Tenetehara-Guajajara
Source: PLoS One. 2018 Sep 5;13(9):e0202394. doi: 10.1371/journal.pone.0202394 (PMC6124704; doi:10.1371/journal.pone.0202394)

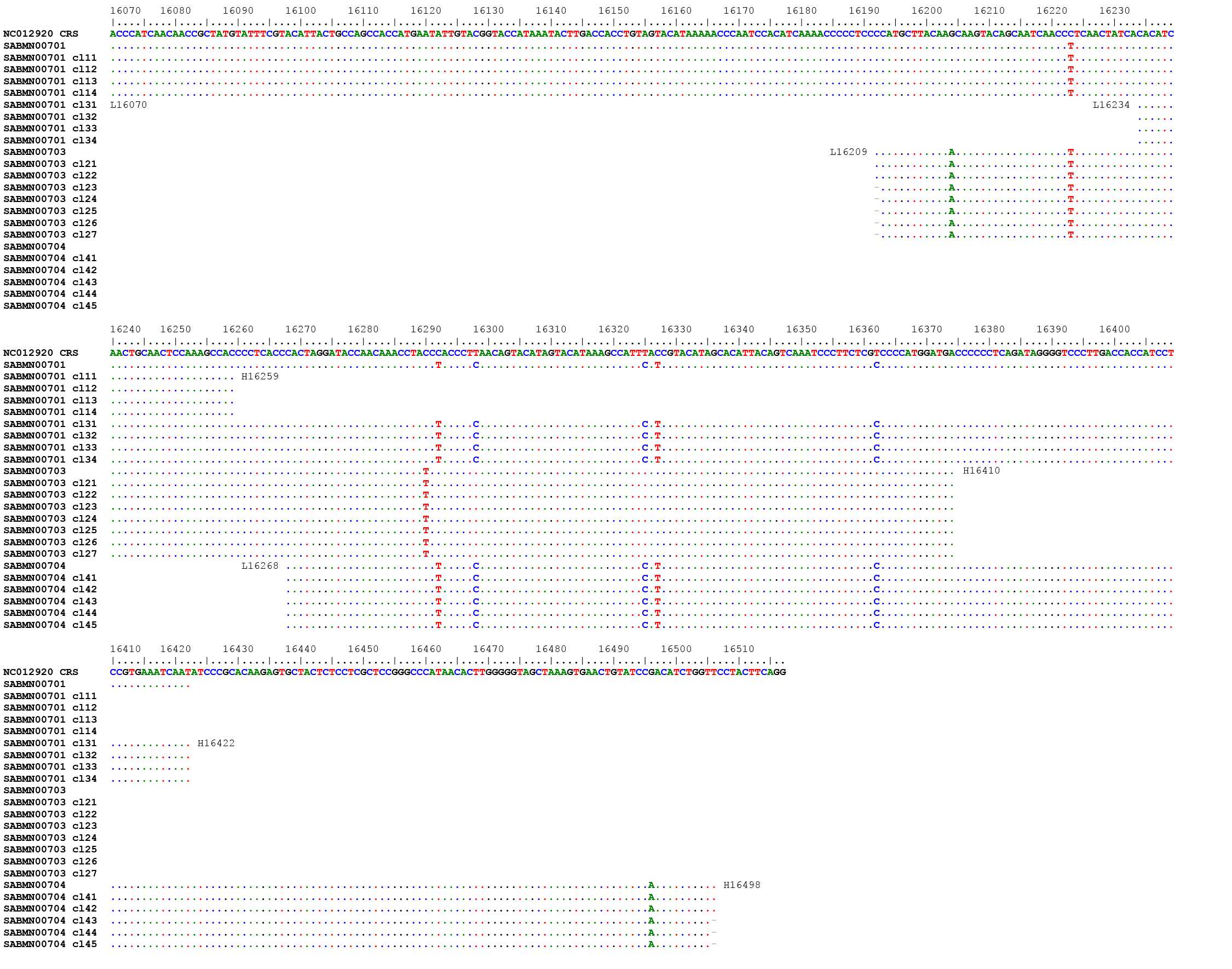

Supplement: S1 Fig — Nomenclature of clones: The first number correspond to mtDNA PCR target (cl1-cl4) and the second to the number of clones generated. Target 1: primers L16070/H16259; 2: L16209/H16410; 3: L16234/H16422; 4: L16268/H16498. (TIF) [file pone.0202394.s002.tif]
